# Defining the incidence and risk factors of colistin-induced acute kidney injury by KDIGO criteria

**DOI:** 10.1371/journal.pone.0173286

**Published:** 2017-03-07

**Authors:** Ryan K. Shields, Rohit Anand, Lloyd G. Clarke, Julie A. Paronish, Matthew Weirich, Hanna Perone, Jake Kieserman, Henry Freedy, Christina Andrzejewski, Hector Bonilla

**Affiliations:** 1 Department of Medicine, University of Pittsburgh, Pittsburgh, Pennsylvania, United States of America; 2 UPMC Mercy Hospital, Pittsburgh, Pennsylvania, United States of America; University of Sao Paulo Medical School, BRAZIL

## Abstract

**Background:**

Acute kidney injury (AKI) remains a treatment-limiting toxicity of colistin. Recently developed clinical practice guidelines from the Kidney Disease: Improving Global Outcomes (KDIGO) group have harmonized definitions of AKI, but have not been widely applied to patients receiving colistin.

**Methods:**

We retrospectively defined AKI by KDIGO definitions among adult patients receiving intravenous colistin for ≥ 3 days. Risk factors for AKI within 48 hours and 7 days of initiating colistin were determined by multivariable logistic regression.

**Results:**

Among 249 patients treated with colistin, rates of AKI were 12% and 29% at 48 hours and 7 days, respectively. At 48 hours, patients in the intensive care unit were at increased risk for AKI. Within 7 days, colistin daily doses >5mg/kg, chronic liver disease, and concomitant vancomycin were independent predictors. Seven percent of patients required renal replacement therapy at a median of 5 days (range: 3–7) following colistin initiation.

**Conclusion:**

Safe use of colistin is promoted by early detection of AKI with KDIGO criteria, avoiding nephrotoxins, and limiting duration of therapy.

## Introduction

Colistin use has resurged due to the emergence of infections caused by extensively drug-resistant (XDR) Gram-negative bacteria. The drug’s efficacy is dependent upon achieving adequate exposures in patients, which requires higher doses than initially prescribed [1]. Ongoing pharmacokinetic (PK) studies have further underscored the importance of a loading dose to mitigate the slow conversion of colistimethate to its active form, colistin [1]. Taken together, these approaches have led to improved PK target attainment [2]; however, the resulting effect on colistin tolerability is largely unknown [3]. Indeed, escalating daily doses of colistin may be associated with a greater risk for acute kidney injury (AKI) [4, 5]. It is unclear if colistin loading doses potentiate this risk, particularly within the first 48 hours of treatment.

AKI remains a treatment-limiting adverse effect of colistin. Colistin-induced AKI appears to be due to the d-aminobutyric and fatty acid components of the drug, which increase cell membrane permeability resulting in cell lysis and acute tubular necrosis [6]. In the modern era, rates of AKI following colistin therapy range from 21–76% [3–5, 7–11]. Rates vary by predisposing conditions and severity of illness, but also criteria used to define AKI [6, 12]. To this end, a recently developed clinical practice guideline from the Kidney Disease: Improving Global Outcomes (KDIGO) group represents a landmark effort in harmonizing AKI definitions [13, 14]. The KDIGO clinical practice guidelines were informed by an exhaustive literature review and recognition that even small changes in absolute serum creatinine levels are associated with adverse outcomes [15, 16]. Indeed, defining AKI with KDIGO criteria is more predictive of in-hospital mortality than the commonly-used RIFLE criteria [17]. By unifying criteria for AKI, clinicians and researchers are armed with improved tools to elucidate risk factors for drug-induced AKI within specific populations. Notably, the KDIGO criteria have been rarely applied to patients receiving colistin [18]. Our objective was to determine the incidence and risk factors for colistin-induced AKI by KDIGO criteria during an era of PK-driven colistin dosing.

## Materials and methods

The protocol was reviewed by the University of Pittsburgh Institutional Review Board (IRB) and determined to meet the necessary criteria for exemption under section 45 of the Code of Federal Regulations. Per local policies and through consultation with the IRB, written patient consent was not required and formal ethical approval was reviewed and waived.

We performed a retrospective, cohort study of adult patients receiving intravenous colistimethate (colistin) for ≥ 3 days at the University of Pittsburgh Medical Center (UPMC) from January 2009 to September 2013. For patients who received more than one course of colistin therapy, only the first was included in the analysis. Those who required hemodialysis (HD) or other types of renal replacement therapy (RRT) at the time of colistin initiation were excluded.

AKI was defined by applying the KDIGO recommendations within 48 hours or 7 days from the initiation of colistin treatment. Specifically, AKI was defined as a ≥0.3 mg/dL increase in SCr from baseline at 48 hours, or a 1.5x increase within 7 days [13, 14]. Creatinine clearance was calculated using the Cockcroft-Gault equation [19]. Continuous and categorical variables were compared with the Mann Whitney U and χ^2^ (or Fisher’s exact) tests, respectively. To determine risk factors for AKI during colistin treatment, we first identified covariates with a *P-*value <0.10 on univariate analysis. Next, we applied multivariable logistic regression to identify independent predictors of AKI using backward selection procedures (STATA SE v.13.1, College Station, TX) Two-tailed *P-*values <0.05 were considered statistically significant.

## Results

Three-hundred and sixty patients received ≥ 3 days of colistin during the study period; 111 patients required RRT prior to colistin treatment and were excluded. Among the remaining 249 patients, the median age was 59 years (inter-quartile range [IQR]: 21–86), 53% (132/249) were male and the median Charlson Comorbidity index was 2 (IQR: 2–6). At the time of colistin initiation, the majority of the patients resided in ICU (64%, 160/249). XDR *Acinetobacter baumannii* was the most commonly targeted pathogen (38%, 94/249), followed by *Pseudomonas aeruginosa* (19%, 48/249), *Klebsiella pneumoniae* (18%, 45/249), and other *Enterobacteriaceae* (5%, 13/249). Sixteen percent of patients were infected by >1 XDR pathogen (39/249) and 4% (10/249) were treated empirically. The median duration of colistin treatment was 8 days (IQR: 5–14). Colistin was employed as part of combination therapy in 86% (215/249) of patients. Twenty-four percent (60/249) of patients received aerosolized colistin as adjunctive therapy and 47% (118/249) received a loading dose. Ninety percent of patients received concomitant nephrotoxic agents, most commonly, vancomycin (56%, 140/249) and loop diuretics (45%, 112/249).

**Fig 1:**
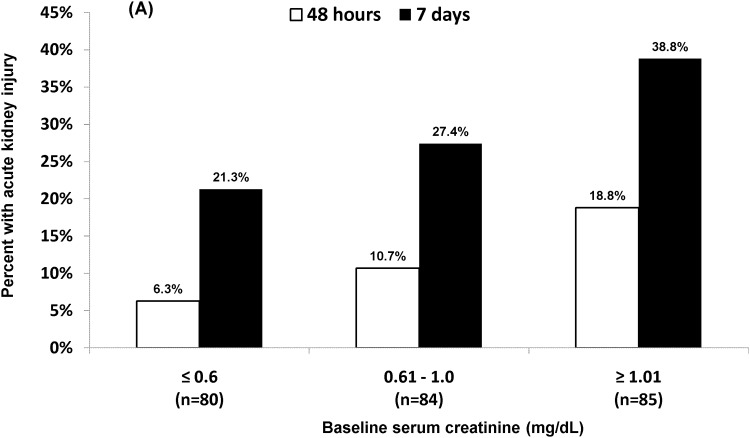
Rates of acute kidney injury by baseline (A) serum creatinine value and (B) creatinine clearance. Note (A). Rates of acute kidney injury were significantly higher for patients with a baseline serum creatinine value ≥1.01 mg/dL compared to ≤0.6 mg/dL at 48 hours (*P* = 0.01) and 7 days (*P* = 0.01).

Criteria for AKI were met within 48 hours and 7 days of colistin initiation in 12% (30/249) and 29% (73/249) of patients, respectively (Table 1). Ninety-eight percent (243/249) and 100% of patients had ≥ 2 SCr values measured within 48 hours and 7 days, respectively. Median baseline SCr values were higher among patients with AKI at 48 hours (1.1 *vs*. 0.8 mg/dL; *P* = 0.006) and 7 days (1.0 *vs*. 0.8 mg/dL; *P* = 0.003). Indeed, rates of AKI were associated with increasing baseline SCr values and decreasing creatinine clearance (Fig 1). Median daily doses of colistin did not differ among patients with or without AKI; however, receipt of a loading dose or a total daily dose >5 mg/kg were more common among patients with AKI at 7 days (Table 1; *P* = 0.039 and 0.044, respectively).

**Table 1:** Factors associated with colistin-induced Acute Kidney Injury (AKI).

Variable	All patients (n = 249)	AKI at 48 hours (≥ 0.3 mg/dL increase in SCr)				AKI within 7 days (≥ 1.5x baseline SCr)			
		AKI	No AKI	Univariate	Multivariate	AKI	No AKI	Univariate	Multivariate
		(n = 30)	(n = 219)	*P-*value	*P-*value (OR, 95% CI)	(n = 73)	(n = 176)	*P-*value	*P-*value (OR, 95% CI)
**Median age (range), years**	59 (18–91)	63 (41–77)	59 (18–91)	0.151		62 (23–81)	57.5 (18–91)	0.034	0.138
**Male sex, no. (%)**	132 (53)	18 (60)	114 (52)	0.414		45 (62)	87 (49)	0.079	0.347
**Caucasian, no. (%)**	198 (80)	21 (70)	177 (81)	0.168		58 (79)	140 (80)	0.987	
**Comorbidities, no. (%)**									
• Chronic respiratory disease	114 (46)	10 (33)	104 (47)	0.206		30 (41)	84 (48)	0.414	
• Congestive heart failure	105 (42)	11 (28)	94 (43)	0.650		34 (47)	71 (40)	0.444	
• Chronic kidney disease	33 (13)	3 (10)	30 (14)	0.575		9 (12)	24 (14)	0.782	
• Chronic liver disease	29 (12)	6 (20)	23 (11)	0.134		14 (19)	15 (9)	0.028	0.011 (3.09, 1.30–7.34)
• Diabetes mellitus	77 (31)	8 (27)	69 (32)	0.591		21 (29)	56 (32)	0.655	
• Obesity (BMI >30)	8 (3)	1 (3)	7 (32)	0.968		3 (4)	5 (3)	0.696	
**Median Charlson Score (range)**	4 (0–11)	3 (0–8)	4 (0–11)	0.353		4 (0–10)	3 (0–11)	0.537	
**Solid-organ transplant recipient, no. (%)**	46 (18)	7 (23)	39 (18)	0.465		17 (23)	29 (16)	0.207	
**Intensive care unit at start of therapy, no. (%)**	160 (64)	26 (87)	134 (61)	0.007	0.024 (3.57, 1.18–10.8)	52 (71)	108 (61)	0.139	
**Requirement for vasopressors, no. (%)**	89 (36)	14 (47)	75 (34)	0.183		29 (40)	60 (34)	0.398	
**Serum albumin < 2 g/dL, no. (%)**	92 (37)	10 (33)	82 (37)	0.662		28 (38)	64 (36)	0.767	
**Median baseline SCr (range)**	0.8 (0.1–3.7)	1.1 (0.4–2.9)	0.8 (0.1–3.7)	0.006	0.115	1.0 (0.2–3.6)	0.8 (0.1–3.7)	0.003	0.094
Median colistin dose in mg/kg/day (range)*	3.45 (0.5–7.69)	3.49 (1.18–6.06)	3.44 (0.5–7.69)	0.751		3.45 (0.85–7.69)	3.48 (0.5–6.56)	0.802	
**Colistin dose > 5mg/kg/day, no. (%)**	37 (15)	6 (20)	31 (14)	0.413		16 (22)	21 (12)	0.044	0.017 (2.58, 1.18–5.61)
**Colistin loading dose, no. (%)**	118 (47)	17 (57)	101 (46)	0.278		42 (58)	76 (43)	0.039	0.284
**Concomitant inhaled colistin, no. (%)**	60 (24)	6 (20)	54 (25)	0.656		19 (26)	41 (23)	0.630	
**Concomitant nephrotoxins, no. (%)**									
• Intravenous contrast dye	36 (14)	3 (10)	33 (15)	0.588		7 (10)	29 (16)	0.159	
• Aminoglycoside	60 (24)	8 (27)	52 (24)	0.726		19 (26)	41 (23)	0.646	
• Vancomycin	140 (56)	21 (70)	119 (54)	0.105		50 (68)	90 (51)	0.012	0.03 (1.98, 1.07–3.66)
• Amphotericin B	13 (5)	4 (13)	9 (4)	0.057	0.088	5 (7)	8 (5)	0.533	
• Calcineurin inhibitor	45 (18)	7 (23)	38 (17)	0.425		17 (23)	28 (16)	0.168	
• Angiotensin converting enzyme inhibitor	12 (5)	0 (0)	12 (5)	0.370		1 (1)	11 (6)	0.189	
• Loop diuretic	112 (45)	13 (43)	99 (45)	0.847		39 (53)	73 (41)	0.085	0.151
• NSAID	44 (18)	2 (7)	42 (19)	0.125		11 (15)	33 (19)	0.488	
**Concomitant antibiotics, no. (%)**									
• Penicillin	83 (33)	15 (50)	68 (31)	0.061	0.075	24 (33)	59 (34)	0.922	
• Cephalosporin	64 (26)	8 (27)	56 (26)	0.898		21 (29)	43 (24)	0.476	
• Carbapenem	184 (74)	22 (73)	162 (74)	0.940		56 (77)	128 (73)	0.515	
• Fluoroquinolone	41 (16)	3 (10)	38 (17)	0.433		11 (15)	30 (17)	0.702	
• Tetracycline	37 (15)	5 (17)	32 (15)	0.785		9 (12)	28 (16)	0.470	
• Macrolide	31 (12)	2 (7)	29 (13)	0.391		5 (7)	26 (15)	0.095	

AKI = Acute kidney injury, BMI = Body mass index, NSAID = Non-steroidal anti-inflammatory drug, SCr = Serum creatinine

* Defined as the average daily dose administered over the first 72 hours of colistin therapy divided by total body weight.

In multivariate analyses (Table 1), after controlling for baseline SCr, residence in the ICU at the time of colistin initiation (OR = 3.57, 95% CI: 1.18–10.8; *P* = 0.024) was an independent predictor of AKI at 48 hours. By 7 days, colistin daily doses >5 mg/kg (OR = 2.58, 95% CI: 1.18–5.61; *P* = 0.017), receipt of concomitant vancomycin (OR = 1.98, 95% CI: 1.07–3.66; *P* = 0.03) and chronic liver disease (OR = 3.09, 95% CI: 1.30–7.34; *P* = 0.011) were associated with AKI. Following colistin initiation, 7% (17/249) of patients required RRT; median time to RRT was 5 days (IQR: 3–7).

## Discussion

Our study represents one of the largest cohorts of patients treated with intravenous colistin reported in the literature, and the largest to define AKI using the KDIGO clinical practice guideline criteria [14, 18]. We have applied these criteria to the first 48 hours and 7 days of colistin treatment, which allows for a dynamic assessment of risk factors early, and over time. Indeed, AKI typically occurs within the first 5–7 days of colistin therapy [4, 20, 21], and is associated with higher rates of mortality compared to late-onset (>7 days) AKI [20]. Here, we found the incidence of AKI at 48 hours and 7 days to be 12% and 29%, respectively. Within the first 48 hours of treatment, colistimethate is slowly converted to its active form, colistin. Administration of a loading dose, results in earlier achievement of steady-state concentrations [1, 22]; however, the incidence and risk factors for AKI within these first 48 hours are unknown. In our analysis, we found ICU residence, but not administration of a loading dose, to be predictive of AKI within 48 hours of initiating colistin. These findings support patient severity of illness as an important determinant of AKI [23]. By 7 days, colistin daily dosage, chronic liver disease and vancomycin co-administration were independent predictors of AKI. Taken together, the data highlight that AKI occurs at variable frequencies among highly dynamic patient populations over time, within whom the interplay between predisposing conditions and acute processes is largely indistinguishable [3]. As such, moving towards consensus definitions, such as those proposed by KDIGO, is imperative. Early detection and treatment of AKI improves patient outcomes, which may be an advantage of KDIGO compared to RIFLE definitions [14].

Risk factors for colistin-induced AKI, by any criteria, are subject to the population being studied, the dose and duration of colistin treatment, and the co-administration of other nephrotoxic agents [6]. At our center, we corroborated the additional hazard of ICU residence [3, 5] and chronic liver disease [5, 24]. Such patients are subject to numerous physiologic changes making them more susceptible to AKI. Critically-ill patients, for example are at greater risk for AKI due to the presence of septic shock and a greater severity of illness [23]. In patients with chronic liver disease, increased nitric oxide and angiotensin II result in hypoperfusion of the kidneys [25, 26]. We also found concomitant vancomycin increases the risk for AKI within 7 days of starting colistin therapy. The association has been linked to the duration of vancomycin therapy [27], and is noteworthy given the ubiquitous use of vancomycin among critically-ill patients, and the potential synergistic activity of colistin-vancomycin combinations against XDR Gram-negative pathogens [28]. Like other nephrotoxins, the requirement for co-administration of vancomycin should be balanced with the additive risk of AKI when using colistin. Prediction models that incorporate concomitant nephrotoxins may be useful in this regard to estimate the risk of nephrotoxicity in individual patients [29]. In managing individual patients, other factors associated with an increased risk for AKI, like older age, pre-existing renal impairment, and underlying diseases, should be evaluated prior to initiating therapy [3].

As colistin dosing strategies evolve, it will be important to weigh the benefits of improved target attainment with the risk of toxicity. To this end, we demonstrated that administration of a colistin loading dose was associated with an increased risk of AKI within 7 days on univariate, but not multivariate analysis. On the other hand, total daily doses exceeding 5mg/kg were independently linked to a higher rate of AKI, consistent with prior studies [4, 29]. These data extend those from recent reports evaluating the safety of colistin loading and PK-optimized dosing strategies [3, 30, 31]. Elefritz and colleagues did not find a significant difference in rates of AKI by RIFLE criteria among 30 patients who received a 5 mg/kg loading dose compared to 42 patients who did not; however rates of AKI were 58% and 50%, respectively [30]. Rates of AKI were lower (44%) among a prospective, observational cohort study of patients who received a loading dose of colistin, followed by PK-driven maintenance doses, but doses were capped at a maximum of 270mg for patients with normal renal function [3]. Most recently, Rigatto and colleagues found a significantly higher rate of renal failure (by RIFLE criteria) among patients who received a loading dose of colistin (77.3% *vs* 23.7%; P≤0.001) though post-hoc analysis showed loading dose patients were older, had more comorbidities, and significantly lower creatinine clearance [31]. Taken together with the current study, the safety of colistin loading doses remains unclear. It is worth highlighting, however, that such approaches are necessary to achieve appropriate serum levels [1]. Importantly, new colistin dosing recommendations advocate for higher daily doses than are currently approved by the US Food and Drug Administration [22, 32]. The impact of these recommendations on rates of AKI will need to be determined in future studies. So too, will the role of renal-protective agents like ascorbic acid, proanthocyanidin, vitamin E, and melatonin [3, 33–35]. Until such data become available, the use of colistin in the clinic requires immediate and continuous monitoring as outlined in the KDIGO guidelines [13].

Like all retrospective studies, ours is not without the limitations that stem from this study design, including the availability of clinical and laboratory data. Though serum creatinine values were readily available, and used to define AKI, future studies may be improved by using more sensitive biomarkers of renal injury [36]. Moreover, we were unable to define AKI by urine output, something that future prospective studies could expand upon. Nonetheless, we defined the rate of colistin-induced AKI using the KDIGO guideline recommendations for the first time at 48 hours and 7 days from the initiation of colistin therapy. In doing so, we have highlighted several patient populations that merit closer monitoring for colistin-induced AKI, namely those with pre-existing renal impairment, chronic liver disease, or treated in the ICU. Concomitant nephrotoxins like, amphotericin B and vancomycin should be used judiciously and whenever possible the duration of colistin therapy should be minimized.
